# Expression of the Genetic Suppressor Element 24.2 (GSE24.2) Decreases DNA Damage and Oxidative Stress in X-Linked Dyskeratosis Congenita Cells

**DOI:** 10.1371/journal.pone.0101424

**Published:** 2014-07-02

**Authors:** Cristina Manguan-Garcia, Laura Pintado-Berninches, Jaime Carrillo, Rosario Machado-Pinilla, Leandro Sastre, Carme Pérez-Quilis, Isabel Esmoris, Amparo Gimeno, Jose Luis García-Giménez, Federico V. Pallardó, Rosario Perona

**Affiliations:** 1 Instituto de Investigaciones Biomédicas CSIC/UAM, Madrid, Spain; 2 CIBER de Enfermedades Raras, Valencia, Spain; 3 Biomedical Research Institute INCLIVA, Valencia, Spain; 4 Department of Physiology, Faculty of Medicine and Dentistry, University of Valencia, Valencia, Spain; University of Newcastle, United Kingdom

## Abstract

The predominant X-linked form of Dyskeratosis congenita results from mutations in *DKC1*, which encodes dyskerin, a protein required for ribosomal RNA modification that is also a component of the telomerase complex. We have previously found that expression of an internal fragment of dyskerin (GSE24.2) rescues telomerase activity in X-linked dyskeratosis congenita (X-DC) patient cells. Here we have found that an increased basal and induced DNA damage response occurred in X-DC cells in comparison with normal cells. DNA damage that is also localized in telomeres results in increased heterochromatin formation and senescence. Expression of a cDNA coding for GSE24.2 rescues both global and telomeric DNA damage. Furthermore, transfection of bacterial purified or a chemically synthesized GSE24.2 peptide is able to rescue basal DNA damage in X-DC cells. We have also observed an increase in oxidative stress in X-DC cells and expression of GSE24.2 was able to diminish it. Altogether our data indicated that supplying GSE24.2, either from a cDNA vector or as a peptide reduces the pathogenic effects of Dkc1 mutations and suggests a novel therapeutic approach.

## Introduction

Telomeres are nucleoprotein complexes located at the ends of linear chromosomes and consist of tandem repeats of simple DNA sequences (TTAGGG in humans) and proteins that interact directly or indirectly with these sequences [1]. Sequence erosion of terminal repeats is inherent to each round of genome replication. The replenishment of the telomeric repeats is accomplished by the extension of their 3′ ends, through a reaction mediated by the telomerase complex [2]. In humans, the active telomerase complex consists of a minimum of three essential components: hTERT, hTR and dyskerin [3]. Besides forming part of the telomerase complex, dyskerin is a pseudouridine synthase component of H/ACA small nuclear RNPs [4], complexes that mediate the conversion of specific uridines (U) to pseudouridine in newly synthesized ribosomal RNAs [5] [6] [7]. Point mutations in dyskerin cause a rare disease named X-linked dyskeratosis congenita (X-DC) [8]. Individuals with X-DC display features of premature ageing, as well as nail dystrophy, mucosal leukoplakia, interstitial fibrosis of the lung and increased susceptibility to cancer [9]. The tissues affected by X-DC, such as bone marrow and skin, are characterized by the high rate of turnover of their progenitor cells.

Telomere shortening prevents the formation of the loop-like structure maintained by a nucleoprotein structure consisting of telomeric DNA and 6 proteins that are together known as shelterin [1]. This capping structure prevents the otherwise exposed ends of different chromosomes from being recognized as double strand breaks (DSBs) by the cell's DNA repair machinery which would result in telomere fusion. When telomeres become critically short or unprotected because of shelterin deficiency, they trigger a DNA damage response (DDR), leading to the activation of an ataxia telangiectasia mutated (ATM) or ataxia telangiectasia and Rad3 related (ATR)-dependent DNA damage response at chromosome ends [10] [11] [12] [13]. 53BP1 is a C-non-homologous-end-joining (C-NHEJ) component and an ATM target that accumulates at DSBs and uncapped telomeres [14] [15]. The binding of 53BP1 close to DNA breaks impacts the dynamic behavior of the local chromatin and facilitates the non-homologous-end-joining (NHEJ) repair reactions that involve distant sites [16]. ATM phosphorylates Chk2 leading to activation of cell cycle checkpoints. Chk2 acts as a signal distributor, dispersing checkpoint signal to downstream targets such as p53, Cdc25A, Cdc25C, BRCA1 and E2F1 [17].

Senescence, initially described as stable cell proliferation arrest, can be induced by telomere shortening and also by activated oncogenes, DNA damage and drug-like inhibitors of specific enzymatic activities [18]. Senescent cells are typically characterized by a large flat morphology and the expression of a senescence-associated-β-galactosidase (SA-β-gal) activity of unknown function. In the nucleus of senescent cells chromatin undergoes dramatic remodeling through the formation of domains of heterochromatin called senescence-associated heterochromatin-foci (SAHF). SAHF contain histone modifications and proteins characteristic of silent heterochromatin such as methylated lysine 9 of histone H3 (H3K9me), heterochromatin protein 1 (HP1), and the histone H2A variant macroH2A.1 [18] [19]. Proliferation-promoting genes such as E2F-target genes (e.g. cyclin A) are recruited into SAHF, dependent on the pRB suppressor protein, thereby irreversibly silencing expression of those genes.

In cultured cells and animal models, telomere erosion promotes chromosomal instability via breakage-fusion-bridge cycles, contributing to the early stages of tumorigenesis. Telomere shortening in Dyskeratosis congenita is associated with a higher risk of some types of cancer such as head and neck squamous cell carcinoma (HNSCC) (mostly tongue), skin squamous cell carcinoma (SCC), anogenital, stomach, esophagus, and lymphomas, as well as myelodysplastic syndrome (MDS) [20] [21] [22] [23]. Altogether, these findings provide direct clinical evidence that short telomeres in hematopoietic cells are dysfunctional, mediate chromosomal instability and predispose to malignant transformation in a human disease.

We previously isolated the peptide GSE24.2, in a screen of cDNAs for those that confer survival ability on cells treated with cisplatin [24]. Intriguingly GSE24.2 turned out to be a short dyskerin fragment containing two highly conserved motifs implicated in pseudouridine synthase catalytic activity. GSE24.2 prevents telomerase inhibition mediated by different chemotherapeutic agents, including cisplatin and telomerase inhibitors. In X-DC cells and WI-38-VA13 cells, GSE24.2 induces an increase in hTERT mRNA levels and the recovery of telomerase activity [24]. Mutations in DKC1 lead to severe destabilization of telomerase RNA (TR), a reduction in telomerase activity and a significant continuous loss of telomere length during growth [25]. When a peptide encoding GSE24.2, was introduced into mutant cells, it rescued telomerase activity and prevented the decrease in TR levels induced by the Dkc1 mutation [26] GSE24.2 was recently approved as an orphan drug for the treatment of Dyskeratosis congenita (EU/3/12/1070 - EMA/OD/136/11).

To obtain more information on the biological activity of this dyskerin fragment we studied its effect on the DNA damage pathway in patient derived X-DC cells and a mouse F9 cell line carrying the A353V mutation in the *Dkc1* gene [26]. This is the mutation most frequently found in patients with X-DC (about 40% of patients) [27] [28] and is localized in the PUA RNA binding domain, the putative site for interaction with hTR. Recently, it has been described that mouse ES cells expressing a small dyskerin deletion, removing exon 15 of *Dkc1*, additionally showed decreased proliferative rate and increased sensitivity to DNA damage [29] that was independent on telomere length suggesting that decreased telomerase activity induced by the mutation in *Dkc1* resulted in induction of DNA damage probably by extratelomeric activity of *Dkc1* gene. Therefore the use of a mouse F9A353V model would allow study the effect of GSE24.2 directly on DNA damage, independently of telomeric elongation. Here we show that human X-DC cells showed both basal DNA damage foci and phosphorylation of ATM and CHK2 together with increased content of heterochromatin. Expression of the GSE24.2 was able to reduce DNA damage in X-DC patient and F9 X-DC mouse cell line models, by decreasing the formation of DNA damage foci. Finally, we also report that expression of GSE24.2 decreases oxidative stress in X-DC patient cells and that may result in reduced DNA damage. These data support the contention that expression of GSE24.2, or related products, could prolong the lifespan of dyskeratosis congenita cells.

## Materials and Methods

### Cell lines and constructs

Dermal fibroblasts from a control proband (X-DC-1787-C) and two X-DC patients (X-DC-1774-P and X-DC3) were obtained from Coriell Cell Repository. GSE24.2, DKC, motif I and motif II were cloned as previously described in the pLXCN vector [24]. PGATEV protein expression plasmid [30] was obtained from Dr. G. Montoya. PGATEV-GSE24.2 was obtained by subcloning the GSE24.2 fragment into the NdeI/XhoI sites of the pGATEV plasmid as previously described [24].

F9 cells and F9 cells transfected with A353V targeting vector were previously described [31] [26]. F9A353V cells were cultured in Dulbecco modified Eagle medium (DMEM) 10% fetal bovine serum, 2 mM glutamine (Gibco) and Sodium bicarbonate (1,5 gr/ml).

### Cell transfection and analysis of gene expression

F9 cells were transfected with 16 µg of DNA/10^6^ cells, using lipofectamine plus (Invitrogen, Carlsbad, USA), according to the manufacturer's instructions. Peptides transfection was performed by using the Transport Protein Delivery Reagent (50568; Lonza, Walkersville, USA) transfection kit. Routinely from 6 to 15 µg were used per 30 mm dish.

#### Antibodies

The source of antibodies was as follow: phospho-Histone H2A.X Ser139 (2577; Cell Signaling), phospho-Histone H2A.X Ser139 clone JBW301 (05-636; Millipore), macroH2A.1 (ab37264; abcam), 53BP1 (4937; Cell Signaling), anti-ATM Protein Kinase S1981P (200-301-400; Rockland), phospho-Chk2-Thr68 (2661; Cell Signaling), Monoclonal Anti-α-tubulin (T9026; Sigma-Aldrich), Anti-8-Oxoguanine Antibody, clone 483.15 (MAB3560, Merck-Millipore). Fluorescent antibodies were conjugated with Alexa fluor 488 (A11029 and A11034, Molecular Probes) and Alexa fluor 647 (A21236, Molecular Probes, Carlsbad, USA)).

### Immunofluorescence and Fluorescence in situ hybridization (FISH) for telomeres

Protein localization was carried out by fluorescence microscopy. For this purpose, cells were grown on coverslips, transfected and fixed in 3.7% formaldehyde solution (47608; Fluka, Sigma, St. Louis, USA) at room temperature for 15 min. After washing with 1x PBS, cells were permeabilized with 0.2% Triton X-100 in PBS and blocked with 10% horse serum before overnight incubation with γ-H2A.X, 53BP1, p-ATM, p-CHK2 antibodies. Finally, cells were washed and incubated with secondary antibodies coupled to fluorescent dyes (alexa fluor 488 or/and alexa fluor 647).

For immuno-FISH, immunostaining of 53BP1 was performed as described above and followed by incubation in PBS 0,1% Triton X-100, fixation 5 min in 2% paraformaldehyde (PFA), dehydration with ethanol and air-dried. Cells were hybridized with the telomeric PNA-Cy3 probe (PNA Bio) using standard PNA-FISH procedures. Imaging was carried out at room temperature in Vectashield, mounting medium for fluorescence (Vector Laboratories, Burlingame, USA). Images were acquired with a Confocal Spectral Leica TCS SP5. Using a HCX PL APO Lambda blue 63×1.40 OIL UV, zoom 2.3 lens. Images were acquired using LAS-AF 1.8.1 Leica software and processed using LAS-AF 1.8.1 Leica software and Adobe Photoshop CS. Colocalization of 53BP1 foci and the PNA FISH probe was quantified in at least 200 cells.

### Telomeric repeat amplification protocol (TRAP) assay

Telomerase activity was measured using the TRAPeze kit [32] (Millipore, Billerica, MA USA) according to the manufacturer's recommendations. TRAP assay activity was normalized with the internal control [24].

### Real-time quantitative PCR

#### RNA isolation and cDNA synthesis

Total cellular RNA was extracted using Trizol (Invitrogen, Carlsbad, USA) according to the manufacturer's instructions. For reverse transcription reactions (RT), 1 µg of the purified RNA was reverse transcribed using random hexamers with the High-Capacity cDNA Archive kit (Applied Biosystems, P/N: 4322171; Foster City, CA) according to the manufacturer's instructions. RT conditions comprised an initial incubation step at 25°C for 10 min. to allow random hexamers annealing, followed by cDNA synthesis at 37°C for 120 min, and a final inactivation step for 5 min. at 95°C.

#### Measurement of mRNA Levels

The mRNA levels were determined by quantitative real-time PCR analysis using an ABI Prism 7900 HT Fast Real-Time PCR System (Applied Biosystems, Foster City, CA). Gene-specific primer pairs and probes for *SOD1* (*SOD Cu/Zn*), *SOD2* (*SOD Mn*), *GPX1 (Glutathione peroxidase 1)* and *CAT* (*Catalase*) (Assay-on-demand, Applied Biosystems), were used together with TaqMan Universal PCR Master Mix (Applied Biosystems, Foster City, USA) and 2 µl of reverse transcribed sample RNA in 20 µl reaction volumes. PCR conditions were 10 min. at 95°C for enzyme activation, followed by 40 two-step cycles (15 sec at 95°C; 1 min at 60°C). The levels of glyceraldehyde-3-phosphate dehydrogenase (*GAPDH*) expression were measured in all samples to normalize gene expression for sample-to-sample differences in RNA input, RNA quality and reverse transcription efficiency. Each sample was analyzed in triplicate, and the expression was calculated according to the 2^−ΔΔCt^ method.

### GSE24.2 peptide production and purification

E. Coli DH5a cells were transformed with pGATEV GSE24.2 and lysates prepared as described [30]. The fusion protein was purified with glutathione-sepharose and purity analyzed by gel electrophoresis. GSE24.2 was obtained from the purified fusion protein by TEV protease digestion according to the manufacturer's recommendations. Typically, over 90% of the fusion protein was cleaved, as determined by SDS-PAGE. The protein was passed twice over a 5 ml Hi-Trap Ni-NTA column to remove the polyhistidine tags, un-cleaved protein, TEV protease and impurities. Synthetic GSE24.2 was obtained from Peptide 2.0 Inc (Chantilly, USA) and purified by HPLC

### Western Blot

Whole-cell extracts were prepared essentially as described previously [33]. Nuclear extracts were obtained as previously reported [24]. Western blotting was performed using standard methods [33]. Protein concentration was measured by using the Bio-Rad protein assay.

### Senescence analysis

Control and X-DC fibroblasts (1×10^4^ cells) were plated onto 6 well plates and fixed after four days to assay the SA-β-gal (Senescence Detection Kit, BioVision, Milpitas, USA). The percentage of senescent cells was calculated in 6 images per sample taken in the bright field microscopy at 100× magnification (Nikon Eclipse TS100 Microscopy, Melville, NY, USA).

### Determination of reactive oxygen species (ROS) content with dihydroethidium

Cells were cultured in 12 chamber plates for 4 days (at confluence). Afterwards cells were washed 2 times with pre-warmed PBS medium, 2 µL/mL of diluted dihydroethidium (Dihydroethidium, D7008-Sigma, St. Louis, USA) was added to the plate. Cells were incubated at 37°C for 20 min. After washing the plate with PBS, medium was replaced, and cells cultured for an additional 1 hour at 37°C. The fluorescence was measured using spectraMAX GEMINIS (Molecular Device, Sunnyvale, USA), with 530 nm of excitation wavelength and 610 nm of emission wavelength. Mean fluorescence intensity (MFI) for each cell line, was normalized by the cellular protein content.

### Measurement of CuZnSOD and MnSOD activity

To determine MnSOD and CuZnSOD activity the cells were treated as described in the Cayman “Superoxide Dismutase Assay kit” (Ann Arbor, USA). After centrifugation at 10,000 g for 10 min, supernatant was used to measure CuZnSOD activity. The mitochondrial pellet was lysed using a lysis buffer compatible with the manufacturer's instructions (10 mM HEPES, pH7.9, 420 mM NaCl, 1,5 mM MgCl_2_, 0,5 mM EDTA, 0.1% Triton X-100) for 20 min on ice. After centrifugation at 12,000 g for 5 min, the supernatant was collected for MnSOD activity assay. Measurements of CuZnSOD and MnSOD activities were performed in a 96 well plate prepared using 3–4 replicates from different cellular extracts for each sample. The final absorbance was measured at 450 nm using a spectrophotometer spectraMAXPLUS 384 (Molecular Devices, Sunnyvale, USA).

### Measurement of catalase activity

The method for measuring the catalase enzymatic activity was based on the reaction of the enzyme with methanol in the presence of hydrogen peroxide to produce formaldehyde. Cells were lysed using freeze (liquid N_2_, 10 s) and thaw (ice, 15 min) procedure repeated three times. After centrifugation of the cell lysate at 13,000 g, for 10 min. at 4°C, supernatants were recovered and quantified using Lowry method. A 96 well plate was prepared using at least 4 replicates for each sample, obtained from different cellular extracts.

Assay reaction consisted in mixing on a 96 well plate: 100 µL of phosphate buffer 100 mM pH 7.0; 30 µL methanol and 20 µL of the sample with the same protein concentration. Then, the reaction was started with 20 µL of 85 mM H_2_O_2_, maintained during 20 min at room temperature and finally stopped using 30 µL of KOH 10 M. The formaldehyde produced reacts with 35 mM purpald reagent dissolved in 0,5 M HCl during 10 min at room temperature. Finally, 10 µL of 0.5% KIO_4_ in KOH 0.5 M were added and the absorbance at the wavelength of 540 nm was measured with spectrophotometer spectra MAXPLUS 384 (Molecular Devices, Sunnyvale, USA).

### Measurement of glutathione peroxidase activity

Gpx activity was measured by using a glutathione peroxidase assay kit (Cayman (Ann Arbor, USA). Briefly, cells were collected and lysed using cold buffer (50 mM Tris-HCl, pH 7.5, 5 mM EDTA and 1 mM DTT) and two freeze-thaw cycles as described above. The lysates were centrifuged at 10,000 g for 15 min at 4°C and the supernatants recovered in fresh tubes. A 96 well plate was prepared using at least 3 replicates for each sample from different cellular extracts. After protein quantification by Lowry method, samples containing 20 µg of total proteins were added to the 96 well plate containing a solution with 1 mM GSH, 0.4 U/mL of glutathione reductase, 0.2 mM NADPH. The reaction was initiated by adding 0.22 mM of cumene hydroperoxide and the reduction of the absorbance was recorded at 340 nm each 1 min during 8 min. The Gpx activity was determined by the rate of decrease in absorbance at 340 nm (1 mU/mL Gpx). Molar coefficient extinction for NADPH was 0.00622 mM^−1^ cm^−1^.

### Statistical analysis

For the statistical analysis of the results, the mean was taken as the measurement of the main tendency, while standard deviation was taken as the dispersion measurement. T-Student was performed. The significance has been considered at *p<0.05, ** for p<0.01 and *** for p<0.001. GraphPad Software v5.0 was used for statistical analysis and graphic representations.

## Results

### 1-Basal and induced DNA damage response in X-DC cells involves 53BP1, ATM and CHK2 and results in increased heterochromatin formation and senescence

It has been previously demonstrated [29] that a pathogenic mutation in murine *Dkc1* causes growth impairment and the enhancement of DNA damage responses after treatment with the chemotherapeutic agent etoposide. In the context of telomeres of normal length, cells with the dyskerin mutation *Dkc1* ^Δ*15*^ (deletion of exon 15) showed increased number of DNA damage foci as observed by detection of p-H2A.X^Ser139^ (γ-H2A.X) foci and activation of the ATM/p53 pathway.

**Figure 1:**
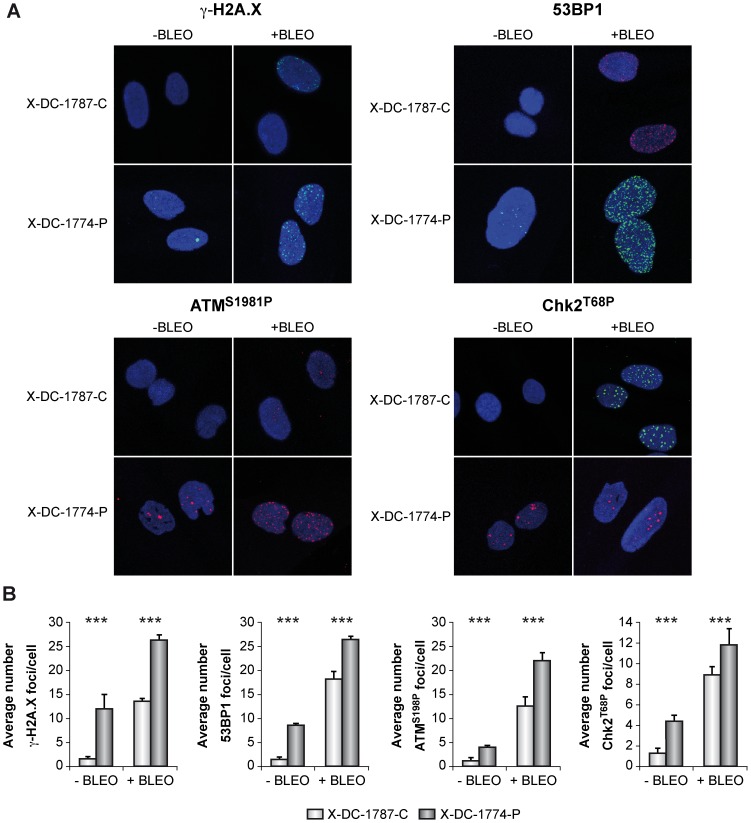
DNA damage signaling in X-DC patient cells. (A) Immunofluorescence staining of DNA damage proteins. Control X-DC-1787-C and patient X-DC-1774-P cells were, either not treated (-Bleo) or treated (+Bleo) with bleomycin (10 µg/ml) for 24 hours, fixed and incubated with antibodies against γ-H2AX, 53BP1, p-ATM or p-CHK1 and secondary fluorescent antibodies. Nuclear DNA was counterstained with DAPI (blue). (B). Quantification of γ-H2A.X foci, pATM, 53BP1 and pCHK2 associated foci in X-DC-1787-C and X-DC-1774-P cells. More than 200 cells were analyzed in each cell line and indicated as the average number of foci/cell. Asterisks indicate significant differences in relation to control cells lines or to untreated cells. Average values and standard deviations of two independent experiments are shown. Experiments were repeated 3 times with similar results.

We have used paired human cell lines (heterozygous carrier and patient) harboring the same mutation in *DKC* gene, responsible for X-DC and studied the DNA damage response pathway. Telomere length of the control cell line (healthy carrier grandmother from X-DC-1774-P patient) was the right length for the age of this control (60 year old and 10.7 kpb). Both basal DNA damage and that produced in response to the DNA damaging agent bleomycin were studied. Our results show that the number of γ-H2A.X-associated foci/cell was dramatically higher in cells obtained from the X-DC-1774-P patient than in the carrier cell line X-DC-1787-C (Fig. 1A and B). When cells were treated with bleomycin, which induces double strand breaks, we found an increase in the number of γ-H2A.X associated foci/cell in both X-DC-1774-P and X-DC-1787-C cells. Although basal DNA damage in X-DC-1774-P was already much higher than that of control cells the increase was similar or even lower to that observed in control cells. We also investigated the presence of 53BP1 foci in these cell lines, since 53BP1 is recruited to DNA-damage associated foci. We found the average number of foci/cell was similar to that observed for γ-H2A.X, higher to that observed in control cells but even if there is an increase after bleomycin treatment, the increase in the number of foci/cell was smaller than control cells ATM protein is also recruited to DNA-damage sites at the chromatin and phosphorylated, we found that X-DC-1774-P cells showed higher number of foci/cell with phosphorylated ATM compared to carrier cells. In bleomycin treated cells both patient and carrier cells showed increased response to DNA damage although similar to what happen with the other indicators of DNA damage the increase observed in X-DC-1774-P was lower than control cells. CHK2 is a protein, substrate of ATM-kinase. We studied the number of cells with phosphorylated CHK2 at Thr68 and found a higher number of foci/cells in X-DC-1774-P in untreated cells, which increases after bleomycin treatment, but such increase is lower than control cells. Altogether these results indicate that basal DNA damage is higher in X-DC patient cells that in mutation carrier cells, in response to bleomycin this increase is not higher in X-DC cells probably due to the high basal damage observed in these cells. Furthermore we have found that the signaling pathway associated with this DNA damage, include at least 53BP1, ATM and CHK2, although we cannot exclude the participation of other proteins.

**Figure 2:**
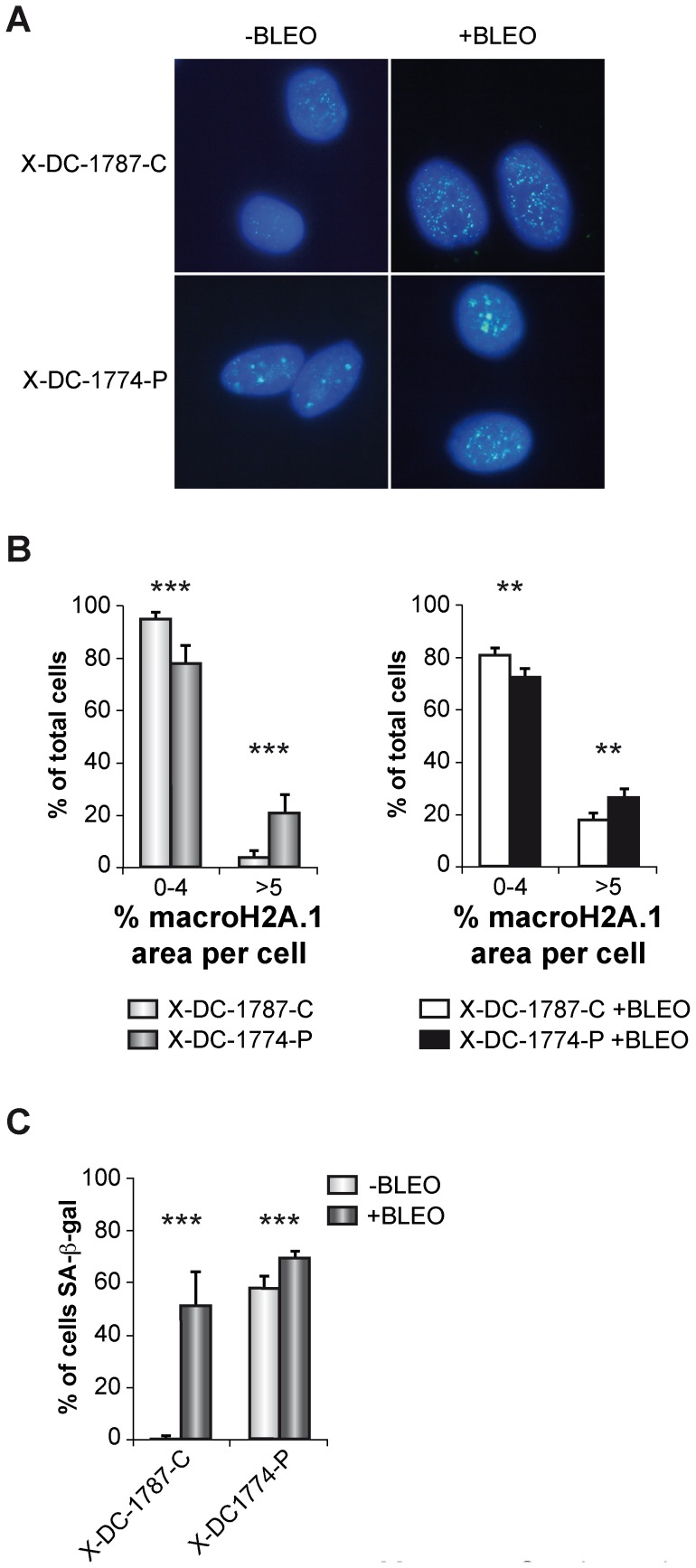
Determination of Histone-macroH2A.1-associated heterochromatin and senescence in X-DC cells. (A) Histone-macroH2A.1-associated heterochromatin detection in X-DC cells. X-DC-1787-C and X-DC-1774-P cells were either not treated (-Bleo) or treated (+Bleo) with bleomycin (10 mg/ml) for 24 hours, fixed and incubated with an antibody against Histone-macroH2A.1 followed by a secondary fluorescence labeled antibody. (B) Quantification of Histone-macroH2A.1-associated heterochromatin. More than 200 cells were analyzed in each cell line and grouped to the area presenting Macro H2A.1 foci per cell. Asterisks indicate significant differences between cells lines. Average values and standard deviations of two independent experiments are shown. (C) SA-β-gal activity in X-DC-1787-C and X-DC-1774-P cells either untreated (-Bleo) or treated (+Bleo) with bleomycin (10 µg/ml). Senescent cells were quantified in 6 images of random regions. Experiments were repeated 3 times with similar results. Asterisks indicate significant differences in response to bleomycin.

In order to verify if X-DC cells harbor an increased heterochromatin content we studied the nuclear distribution of histone-macroH2A.1-associated heterochromatin in X-DC-1774-P and X-DC-1787-C cells, both in basal conditions and after bleomycin treatment (Fig. 2A). X-DC-1774-P cells already showed an average of 20% of the nuclear area with positive expression for macroH2A.1, and after bleomycin treatment we detected an increase up to 30% (Fig. 2B). X-DC-1787-C cells showed a very low expression in basal conditions that increases to almost 20% after bleomycin treatment (Fig. 2C). These data indicated that X-DC patient cells show extensive areas of heterochromatin that further increased in response to bleomycin. Thus, both basal and induced DNA damage may trigger a relevant silencing of gene expression in these cells. Almost 60% of X-DC-1774-P cells were positive for the senescence SA-β-gal activity that increases to almost 70% after bleomycin treatment. X-DC-1787-C cells showed low expression of SA-β-gal that also increases further after bleomycin treatment.

### 2- DNA damage is localized in telomeres in X-DC cells

**Figure 3:**
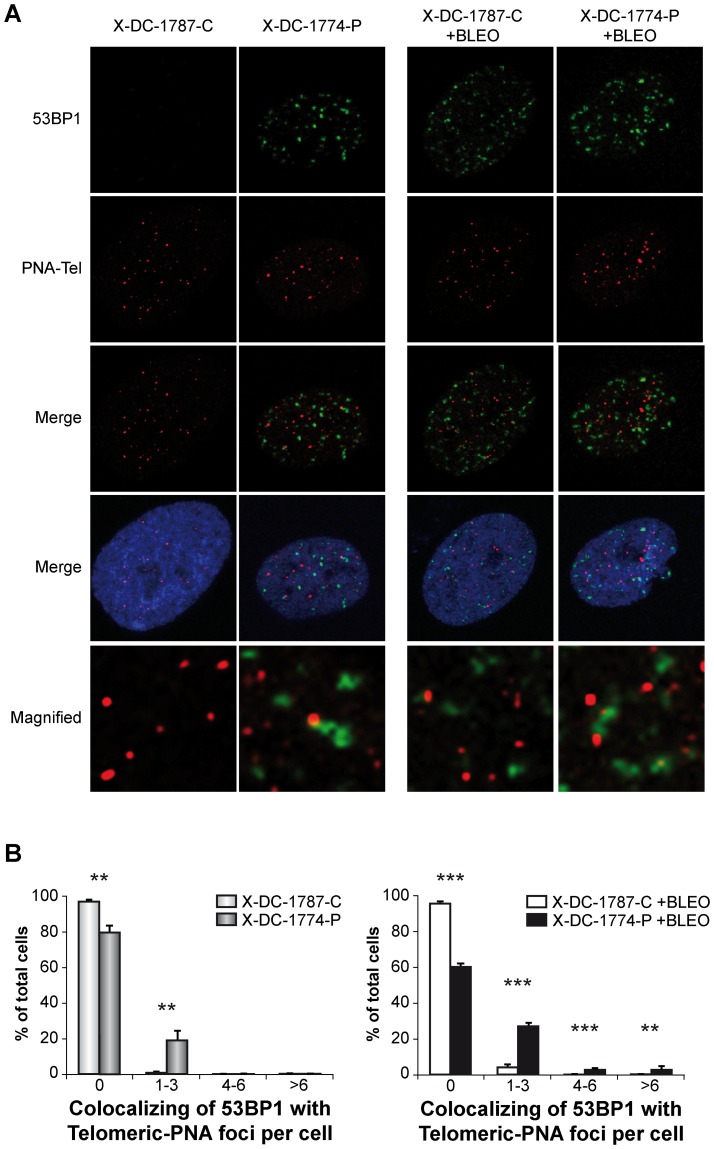
Localization of 53BP1 foci to telomeres in X-DC patient cells. X-DC-1787-C and X-DC-1774-P cells untreated (-Bleo) or treated (+Bleo) with bleomycin (10 µg/ml) and incubated with γ-H2A.X and PNA-FISH probe. (A) Colocalization of 53BP1 foci (green) and telomeres as identified by hybridizing with a PNA-FISH probe (red). DNA was counterstained with DAPI (blue). Magnified views of merged images showing details of the colocalization are shown in the two lower series of panels (B) Colocalized 53BP1 foci and PNA-FISH probe at telomeres was quantified. More than 200 cells were analyzed in each cell line in an experiment performed three times with similar results. Asterisks indicate significant differences in relation to different cell lines.

Since telomere length is greatly diminished in X-DC patient cells we investigated if DNA damage was enriched at telomeres, both in basal conditions and after DNA damage induction. In order to investigate this, we combined a PNA FISH probe as a telomere marker, and 53BP1 for DNA damage detection. The results showed that there was a high association of damaged DNA at the telomeres in X-DC-1774-P cells that was not found in carrier X-DC-1787-C cells (Fig. 3A). Furthermore the increase in DNA damage observed after bleomycin treatment (Fig. 1B) was strongly associated with telomeres in X-DC-1774-P cells in contrast to X-DC-1787-C cells (Fig. 3) indicating the relevance of telomere shortening in the response to DNA damage in X-DC patient cells.

### 3-Expression of GSE24.2 impairs the induction of γ-H2A.X foci after DNA damage

**Figure 4:**
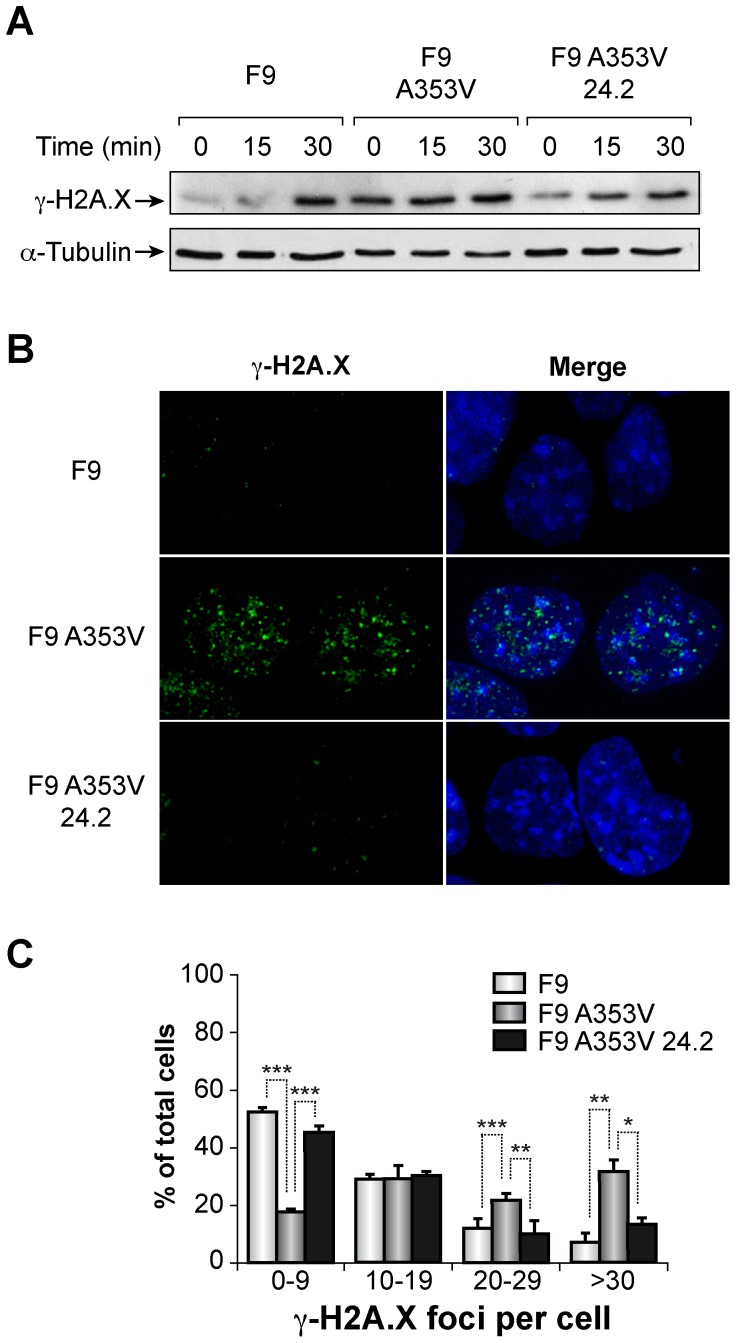
F9A353V cells show enhanced, basal and bleomycin induced, DNA damage response. (A) F9, F9A353V and F9A353V cells transfected with GSE24.2 (10 µg DNA per million cells). F9A353V 24.2 cells were treated with bleomycin (10 µg/ml). After 0, 15 or 30 minutes of treatment cells were lysed and the experiment analyzed by western blot with antibodies against γ-H2A.X or α-tubulin as a loading control. (B) Immunofluorescence staining of γ-H2A.X (green) in F9, F9A353V and F9A353V 24.2 cells (10 µg DNA per million cells). Nuclear DNA was counterstained with DAPI (blue). (C) Quantification of γ-H2AX foci in F9, F9A353V or F9A353V 24.2 cells. More than 200 cells were analyzed in each cell line and grouped to the number of γ-H2A.X foci observed per cell. Experiments were repeated 3 times with similar results. Asterisks indicate significant differences in relation to different cell lines.

Since F9 cells represent a good model system to study DNA damage responses as previously demonstrated (29), we used them in order to investigate if the expression of GSE24.2 could modify the activation of the DNA damage response. Therefore, we transfected F9A353V and control F9 cells [26] with the GSE24.2 expression plasmid and treated them either with bleomycin or etoposide, a topoisomerase inhibitor known to induce DNA double-stranded breaks. We found that, as expected, bleomycin treatment induced γ-H2A.X in both cell lines (Fig. 4A). However, the basal level of γ-H2A.X was much higher in F9A353V cells than in F9 cells expressing the WT dyskerin, indicating that the mutation renders the cells more susceptible to DNA damage. In the presence of the GSE24.2 F9A353V, γ-H2A.X decreased to values very similar to those observed in F9 cells in both, basal and bleomycin-induced levels. Similar results were obtained in etoposide-treated cells (data not shown). We next investigated the presence of γ-H2A.X containing foci in basal conditions and the results confirmed those obtained in the western blot studies (Fig. 4B and 4C). Most F9 cells showed very few foci; the number increased in F9A353V cells but was reduced at similar level to those of F9 cells when the mutant cells were transfected with GSE24.2. Altogether, the results indicated that the expression of GSE24.2 decreases the DNA damage produced by the dyskerin mutation.

**Figure 5:**
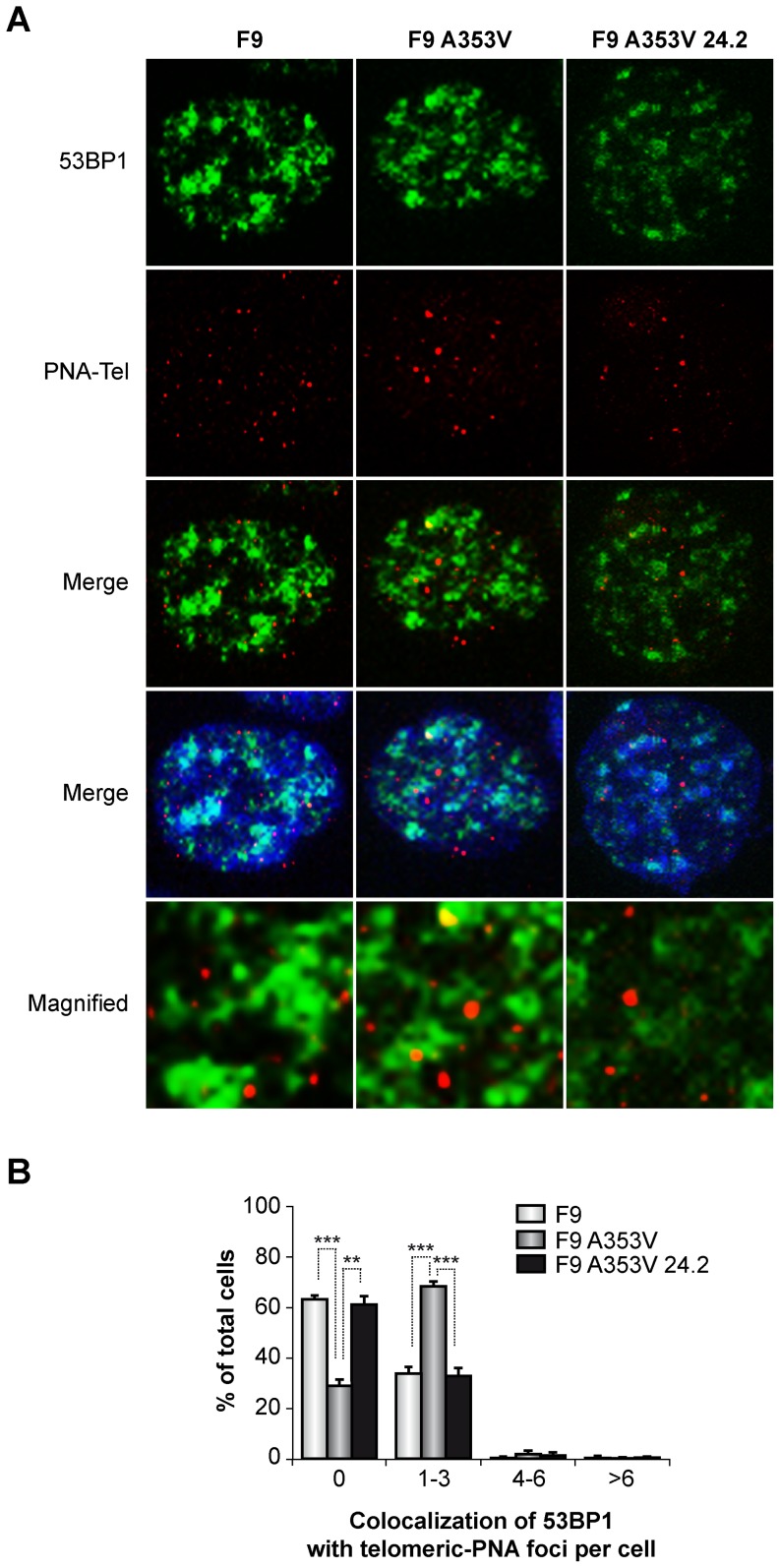
Localization of 53BP1 foci to telomeres in F9A353V cells. F9, F9A353V and F9A353V cells transfected with GSE24.2 (F9A353V 24.2) (F9 cells were treated with bleomycin,10 µg/ml for 24 hours) and incubated with 53BP1 antibodies and with a PNA-FISH probe. (A) Colocalization of 53BP1 foci (green) and PNA-FISH probe that identified telomeres (PNA-Tel, red). DNA was counterstained with DAPI (blue). Magnified views of merged images showing details of the colocalization are shown in the lower panels. (B) Quantification of the colocalization of 53BP1 foci and telomere signals shown in panel A. More than 200 cells were analyzed in each cell line and grouped to the number of 53BP1 foci associated to telomeres (PNA-Tel) per cell. Experiments were repeated 3 times with similar results. Asterisks indicate significant differences in relation to different cell lines.

Afterwards, we investigated if the increased DNA damage in F9A353V cells was enriched at the telomeres (as already found in X-DC patient cells, Fig. 3) and also whether the protection from DNA damage induced by GSE24.2 also applies to damage at the telomeres. We use combined immunological detection of 53BP1 and PNA-FISH probe. The results (Fig. 5A and 5B) indicated that F9A353V cells have a stronger association of 53BP1 to the telomeres than in F9 cells treated with bleomycin, up to 60%. However in F9A353V cells transfected with the GSE24.2 there is little association of 53BP1 foci at the telomeres (30% 1–3 53BP1 foci per cell). These results indicate that the elevated DNA damage response found in F9A353V cells is probably caused by defects at the telomeres induced by the *Dkc1* mutation, in agreement with the results obtained in *Dkc1* ^Δ*15*^ MEF cells. Interestingly, expression of GSE24.2 reverted the telomere damage in F9A353V cells, indicating its biological importance in the reversion of the mutant *Dkc1* phenotype.

### 4-Treatment of X-DC cells with GSE24.2 peptide rescues DNA damage

**Figure 6:**
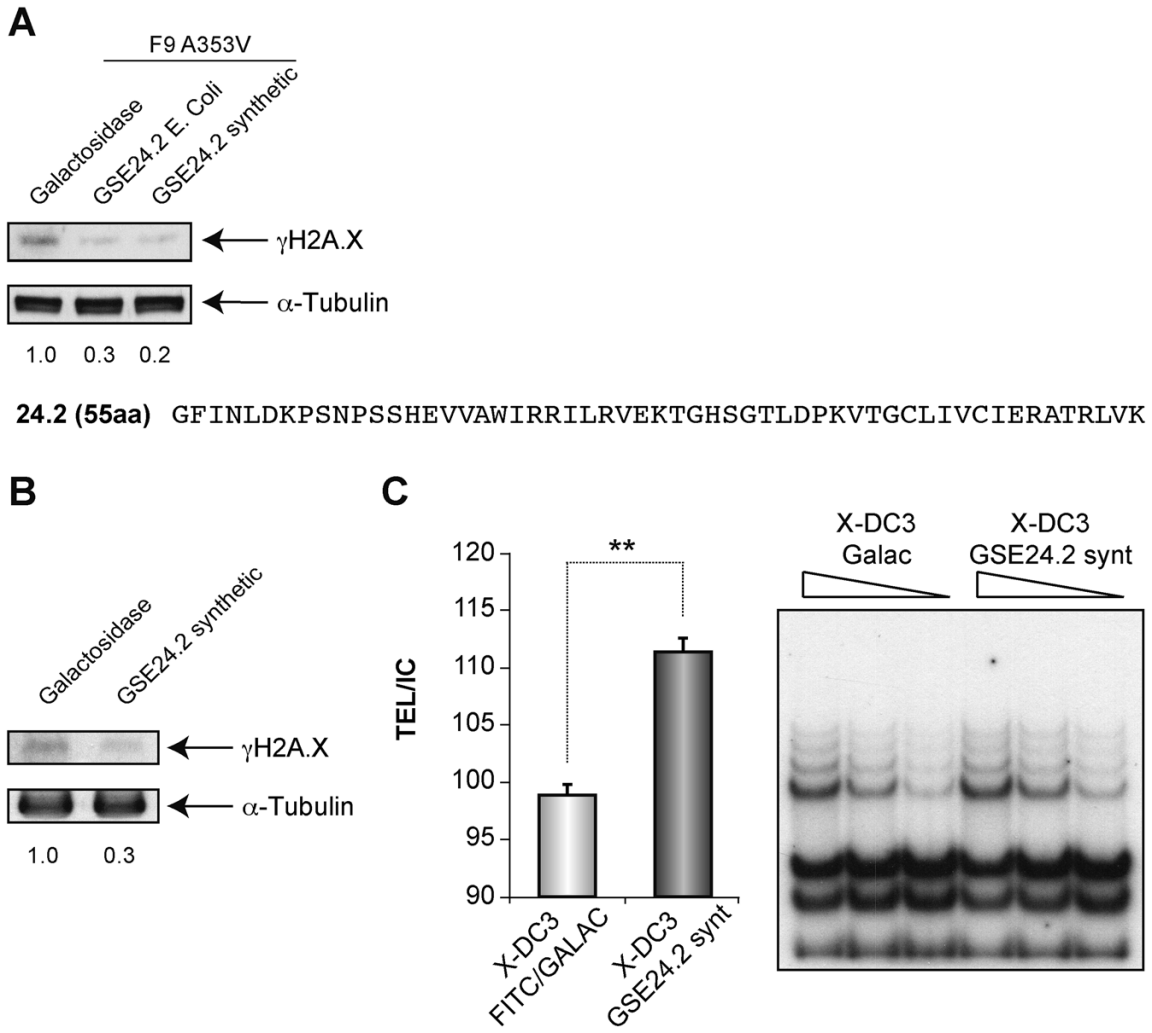
Activity of the GSE24.2 peptide expressed in bacteria or chemically-synthesized. (A) F9A353V cells were transfected with 15 µg of β-galactosidase as a control (galactosidase), or GSE24.2 purified from E. Coli (GSE24.2E.coli) or obtained by chemical synthesis (GSE24.2 synthetic). After 24 hours cells were lysed and the levels of γ-H2AX and α-tubulin determined by western blot. The values at the bottom were obtained after quantification of the blot and show the ration between expression levels of γ-H2AX and α-tubulin in each line and referred to those found in β-galactosidase transfected cells. (B) Same experiment described in A, performed in X-DC3 cells transfected with β-galactosidase or chemically synthesized GSE24.2. (C) Reactivation of telomerase activity by chemically synthesized GSE24.2. X-DC3cells were transfected with β-galactosidase or chemically synthesized GSE24.2 and telomerase activity determined by TRAP assay (right). Different amounts extract were used for each TRAP assay as indicated. The activity was quantified by evaluating the intensity of the bands in relation with the internal control (TEL/IC) (left panel). The values for GSE24.2 transfected cells were referred to the β-galactosidase transfected cells. The experiments were repeated at least three times with similar results. Asterisks indicate significant differences between the two different transfected peptides.

We have previously reported that the GSE24.2 peptide purified from bacteria was able to increase telomerase activity in F9A353V cells [26] therefore we next tested if the activity of the GSE24.2 peptide either purified from E-coli or chemically synthesized reduced the DNA damage. We found that the levels of γ-H2A.X in F9A353V cells decreased after transfecting this peptide (Fig. 6A) either obtained from bacteria or chemically synthesized to 30 and 20%, respectively. Moreover the synthetic peptide also decreased the DNA damage in X-DC3 cells (DKC1 mutated lymphocytes) by 30% (Fig. 6B). This decrease in DNA damage correlated well with the ability of the synthetic peptide to increase telomerase activity in these cells (Fig. 6C).

### 5- Oxidative stress in X-DC cells is decreased by expression of GSE24.2

**Figure 7:**
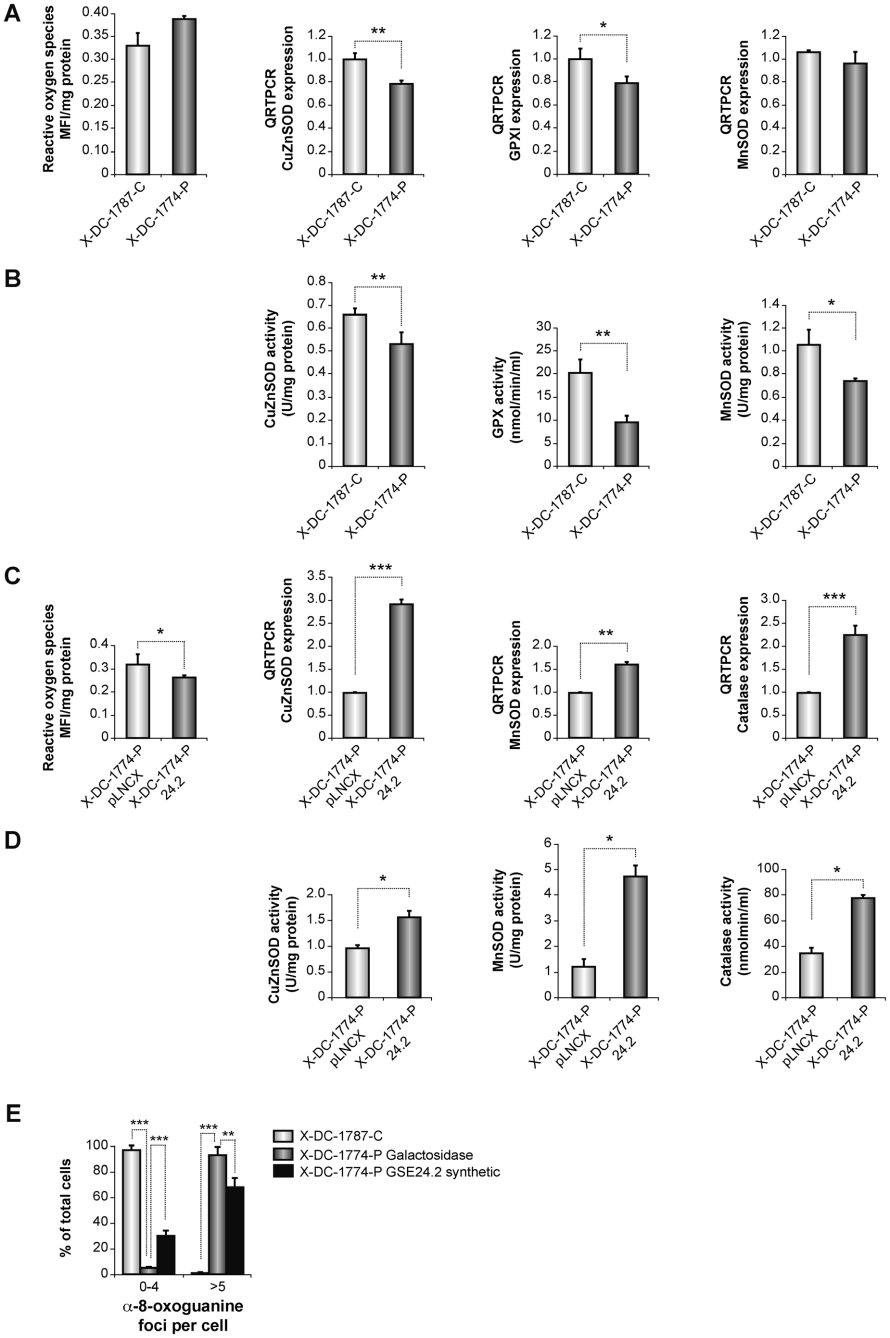
Oxidative stress analysis in X-DC fibroblasts after GSE24.2 transfection. (A) ROS levels were determined in fibroblasts from the carrier DC1787, and fibroblasts from the patient X-DC1774-P. Levels were determined using the fluorescent probe dihydroethidium in confluent cells (left panel). RNA expression was determined for CuZnSOD, MnSOD, and GPX1 by qRT-PCR (A right panels). B) Enzymatic activities of CuZnSOD,MnSOD, and Glutathione peroxidase 1 were also determined. C) ROS levels were studied in X-DC1774-P fibroblasts (expressing pLNCX vector) and X-DC-1774-P cells expressing GSE24.2 (X-DC1774-PGSE24.2, left panel). Cu/ZnSOD, MnSOD, and catalase expression levels were determined by qRT-PCR. D) Cu/ZnSOD, MnSOD, and catalase activities in confluent pLNCX and 24.2 cells are shown in left panels. E) X-DC1774-P and X-DC1774-PGSE24.2, cells were transfected with GSE24.2 synthetic peptide and levels of 8-oxoguanine studied by immunofluorescence. The 8-oxoguanine foci signal was expressed as the average number of foci/cell in 200 cells. Results are expressed as mean ± standard deviation from three independent experiments. Statistical significance is expressed as (*) p<0.05.

Oxidative stress is one of the causes of DNA damage producing both single-strand breaks (SSBs) and double-strand breaks (DSBs). SSBs are the result from the interaction of hydroxyl radicals with deoxyribose and subsequent generation of peroxyl-radicals. These reactive oxygen species (ROS) are then responsible for nicking phosphodiester bonds that form the backbone of each helical strand of DNA [34]. To clarify the presence of higher oxidation levels in X-DC cells we have studied ROS levels, and the expression of antioxidant enzymes CuZn (SOD1) and Mn (SOD2) superoxide dismutase, glutathione peroxidase 1 (GPX1) and their corresponding enzymatic activities in X-DC-1787-C and X-DC-1774-P cell lines. Levels of ROS were elevated in X-DC-1774-P cells compared with X-DC-1787-C carrier cells and also higher than in GM03348, an age-matched cell line from a healthy subject (data not shown). In agreement with this result we found a decrease in gene expression levels of the antioxidant enzymes CuZnSOD and MnSOD and GPX1 when compared the X-DC-1774-P to the carrier cell line (Fig. 7A). We also determined the activity of the three enzymes with decreased expression in the X-DC-1774-P cells that also showed decreased activity in agreement with the gene expression data (Fig. 7B).

In order to investigate if expression of GSE24.2 was able to overcome the increased oxidative stress found in X-DC-1774-P cells, we expressed in this cell line either pLNCX-GSE24.2 or the empty vector (pLNCX). The results indicated that X-DC-1774-P cells expressing GSE24.2 showed lower levels of ROS. We also studied the expression levels of CuZnSOD, MnSOD and catalase in both cell lines and found that expression levels of these antioxidant enzymes were higher in X-DC-1774-P-24.2. When the corresponding protein activities were analyzed, we observed an increase in CuZnSOD, MnSOD and catalase activities (Fig. 7C) in X-DC-1774-P-24.2 when compared with the empty vector transfected cells. Altogether, the data indicated that the observed decrease in oxidative stress in X-DC cells expressing GSE24.2 should contribute to protect these cells from DNA damage. We finally investigated if treatment with the GSE24.2 synthetic peptide was also able to induce a decrease in oxidative DNA damage. We transfected X-DC-1774-P cells with the GSE24.2 synthetic peptide and evaluated the levels of 8-oxoguanine by immunofluorescence (Fig 7E). The results showed that indeed the synthetic GSE24.4 reduced the signal obtained with 8-oxoguanine-antibody.

## Discussion

We have previously reported that expression of a *dyskerin* internal peptide (GSE24.2) reactivates telomerase activity in cells that are deficient in this activity by increasing TERT and TERC levels [24] We have also reported that expression of GSE24.2 increases TR levels by stabilizing this RNA [26]. Because of this activity GSE24.2 has been recently approved as an orphan drug by EMA for the treatment of Dyskeratosis congenita. We have now studied the role of GSE24.2 in the DNA damage response of X-DC patient cells in an effort to better understand the mechanism of GSE24.2 action in X-DC. We studied several proteins involved in the DNA-damage response and found, as other authors have [35] [36], that X-DC patient cells presented higher levels of DNA damage associated foci detected by γ-H2A.X and 53BP1 and to a lesser extent p-ATM and p-CHK2. We also found increased levels of DNA damage in response to bleomycin that was more evident when we studied -H2A.X, p-ATM and p-CHK2 associated foci as previously described in mice (29) but this increase was not higher than that obtained in control cells probably because X-DC cells already have massive damage in basal conditions. Previous reports described increased levels of DNA damage in DC cells harboring mutations in *DKC1*, *TERC* or *TERT*. However in fibroblasts and lymphocytes from these patients the response to induced DNA-damage was not increased [35] in contrast to another study [29]. We have here used X-DC patient cells which exhibited short telomeres, p53 activation and senescence [37]. Indeed, a high level of DNA damage, both at basal and induced by bleomycin, was observed at telomeres suggesting that the shortening of telomeres in these cells induces further damage by preventing repair. Dysfunctional telomeres trigger a DNA damage response most likely because they are too short to adopt the normal t-loop structure needed to form the telomere with correctly ordered shelterin components. Recruitment of histone-macroH2A.1 has been associated to heterochromatin and senescent associated foci (SAHF) [18] [19]. We found that both senescence and macroH2A.1 associated-foci are increased in X-DC patient cells and also that bleomycin treatment increases these values, suggesting that the impairment in the repair of DNA lesions in X-DC cells likely contributes to the senescent phenotype.

Using the *in vitro* generated *Dkc1* mutant F9A353V cells we have found, in agreement with our previous results (and also [29]), that these cells showed increased DDR compared with F9 cells, both in the steady state and when treated with bleomycin or etoposide. Other *Dkc1* mutations such as *Dkc1* ^Δ*15*^ have been shown to accumulate DNA damage indicating that DC cells have cellular defects even in the context of long telomeres [29]. We previously reported that an internal fragment of Dyskerin, the peptide GSE24.2 induces an increase in telomerase activity in X-DC cells [24]. Now we are showing that expression of GSE24.2 is able to induce protection against DNA damage. Furthermore, the repair of pre-existing DNA lesions should also take place at telomeres in F9A353V cells as shown by the decrease in 53BP1 and PNA-FISH telomeric colocalization (Fig. 5B). Interestingly, the observed decrease in DNA damage mediated by GSE24.2 expression in F9A353V cells, also occurs when we used either bacterially produced or chemically synthesized peptide, reinforcing the idea that GSE24.2 reactivates telomerase activity, by acting directly at the telomeric DNA [26] and/or changing telomere folding. According with these results the transfection of the GSE24.2 synthetic peptide into X-DC3 human patient lymphocytes resulted in both increased telomerase activity and decreased DNA damage. On the other hand the consequences of A353V-X-DC mutation on DNA damage resemble to those found in cells with mutations in *Tin2* and *Pot1*, which are structural components of telomeres [38] [39].

Diseases with telomerase deficiency are linked to oxidative stress. Elevated levels of the lipoperoxide malondialdehide (MDA) [40], and MDA-DNA adducts have been reported in rare degenerative diseases [41] and in aging [42]. In addition, oxidative stress conditions caused by H_2_O_2_ increased the rate of telomere shortening in fibroblasts from ataxia-telangiectasia patients [43]. Furthermore, increased accumulation of ROS is involved in decreased cell growth in a *DKC^Δ15^* mouse model [36], though there is very little information about oxidative stress in human X-DC cells. Interestingly, the existence of oxidative stress in lymphocytes from patients with an autosomal dominant form of DC with mutations in *TERC* has been recently reported [44]. To further increase the characterization of the oxidative stress profile in X-DC we characterize the levels of ROS and the expression and activity of the main antioxidant enzymes. We found in X-DC-1774-P an increase in ROS levels and a decrease in the expression and activity of antioxidant enzymes in patient cells when compared to carrier cells. Interestingly expression of GSE24.2 results in an increase in SOD1, SOD2 and catalase expression that might decrease ROS levels in X-DC-1774-P 24.2 cells. Different groups [45] [46] [47] [48] have reported decreased cellular ROS levels in stressed hTERT over-expressing cells, demonstrating that telomerase re-expression contributes to decrease oxidative stress [49]. The work by Westin et al. demonstrated that the reduction of the levels of superoxide in DC cells was not dependent of the localization of TERT in the mitochondria, but also p53/p21^WAF/CIP^-dependent process in the context of telomere shortening in cells from DC patients. Therefore, our findings reinforce the notion that increased telomerase activity [24] and repair of DNA damage at telomeres induced by GSE24.2 is concomitant with a decrease in oxidative stress in X-DC cells. Alternatively the decreased DNA damage detected by γ-H2A.X, might corresponds to decreased oxidative damage, in agreement of our results evaluating the levels of 8-oxoguanine that decreased after transfection of the GSE24.2 synthetic peptide.

**Figure 8:**
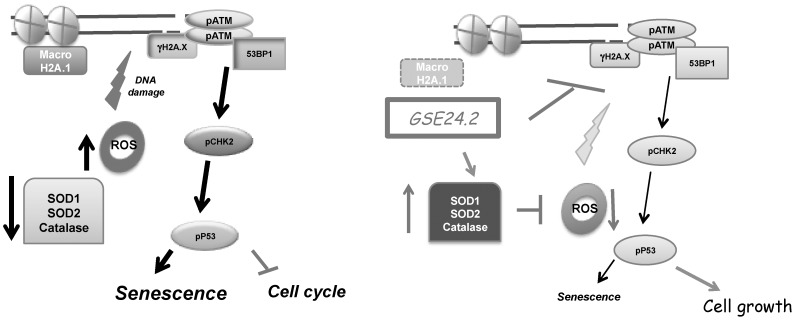
Proposed biological activity of GSE24.2 on DNA damage and oxidative stress. Dyskeratosis congenita cells display high basal DNA damage detected by increased γH2AX, p-ATM, p-CHK2 and 53BP1 foci. Additionally there are increased levels of ROS, and decreased expression and activity of antioxidant enzymes resulting in higher oxidative damage and senescence (left panel). GSE24.2 peptide increases expression of antioxidant enzymes and as a consequence decreased ROS levels (right panel). In parallel there is increased telomerase activity that may help to decrease global and telomeric DNA damage. Globally these two activities of GSE24.2 might result in increased viability and growth of DC cells (26).

In summary our results show that, GSE24.2 attenuates the impact of the *DKC1* mutations on DNA damage and its incidence on telomeres (Fig. 8). Furthermore, oxidative stress decreases in GSE24.2 expressing cells, and this should contribute to decrease the rate of DNA damage and therefore enable restoration of cell cycle progression. Indeed, we have previously shown that expression of GSE24.2 X-DC fibroblasts restores proliferation [26].

Since GSE24.2 has been approved as an orphan drug for the treatment of DC, the results presented here indicate that expression of GSE24.2 may form the basis of a useful and safe therapeutic strategy for X-DC patients either by using it as a permanent or as a temporal telomerase activator. These results indicate that GSE24.2 expression has a broad effect on DC cells, reducing oxidative stress and DNA damage in addition to reactivating telomerase activity. All these protective effects could cooperatively contribute to increase DC cells survival and proliferation [26] and give further support to the recent approval of GSE24.2 as an orphan drug for DC treatment.
